# The Comparability of Anti-Spike SARS-CoV-2 Antibody Tests is Time-Dependent: a Prospective Observational Study

**DOI:** 10.1128/spectrum.01402-21

**Published:** 2022-02-23

**Authors:** Thomas Perkmann, Patrick Mucher, Nicole Perkmann-Nagele, Astrid Radakovics, Manuela Repl, Thomas Koller, Klaus G. Schmetterer, Johannes W. Bigenzahn, Florentina Leitner, Galateja Jordakieva, Oswald F. Wagner, Christoph J. Binder, Helmuth Haslacher

**Affiliations:** a Department of Laboratory Medicine, Medical University of Vienna, Vienna, Austria; b Department of Physical Medicine, Rehabilitation and Occupational Medicine, Medical University of Vienna, Vienna, Austria; Labcorp

**Keywords:** SARS-CoV-2, agreement, serology, time-dependency, vaccination

## Abstract

Various commercial anti-Spike SARS-CoV-2 antibody tests are used for studies and in clinical settings after vaccination. An international standard for SARS-CoV-2 antibodies has been established to achieve comparability of such tests, allowing conversions to BAU/mL. This study aimed to investigate the comparability of antibody tests regarding the timing of blood collection after vaccination. For this prospective observational study, antibody levels of 50 participants with homologous AZD1222 vaccination were evaluated at 3 and 11 weeks after the first dose and 3 weeks after the second dose using two commercial anti-Spike binding antibody assays (Roche and Abbott) and a surrogate neutralization assay. The correlation between Roche and Abbott changed significantly depending on the time point studied. Although Abbott provided values three times higher than Roche 3 weeks after the first dose, the values for Roche were twice as high as for Abbott 11 weeks after the first dose and 5 to 6 times higher at 3 weeks after the second dose. The comparability of quantitative anti-Spike SARS-CoV-2 antibody tests was highly dependent on the timing of blood collection after vaccination. Therefore, standardization of the timing of blood collection might be necessary for the comparability of different quantitative SARS-COV-2 antibody assays.

**IMPORTANCE** This work showed that the comparability of apparently standardized SARS-CoV-2 antibody assays (Roche, Abbott; both given in BAU/mL) after vaccination depends on the time of blood withdrawal. Initially (3 weeks after the first dose AZD1222), there were 3 times higher values in the Abbott assay, but this relationship inversed before boosting (11 weeks after the first dose) with Roche 2 times greater than Abbott. After the booster, Roche quantified ca. 5 times higher levels than Abbott. This must be considered by clinicians when interpreting SARS-CoV-2 antibody levels.

## INTRODUCTION

Infectious diseases continue to pose a significant challenge for humanity, as the severe acute respiratory syndrome coronavirus 2 (SARS-CoV-2) pandemic has again demonstrated (1). Nevertheless, in contrast to the past, diagnostic, therapeutic, and preventive strategies are now being developed at an unprecedented rate to address these pandemic challenges. Among all these strategies, however, the one that stands out is the vaccination against SARS-CoV-2. Using new technologies and extensive knowledge on active immunization against numerous pathogens, highly efficient vaccines have been developed and applied within a few months (2).

The vaccination aims to induce a SARS-CoV-2 specific immune response analogous to a previous infection and thus should protect against disease or even better protect against infection. The simplest way to objectify an immune response is to measure the specific antibodies elicited by an infection or vaccine (3). Thus, SARS-CoV-2 antibody tests can be used to confirm known prior infections or detect unreported infections in seroprevalence surveys (4, 5). For this purpose, different antigens are used, which can be divided into two classes: SARS-CoV-2 nucleocapsid-specific antibodies and antibodies directed against the spike protein (6). The latter antibodies, which are formed against components of the virus surface spike protein, are induced by all COVID vaccines currently in use, making them an ideal surrogate for the immune response after vaccination (7).

The need to develop quantitative assays to detect vaccine-induced antibodies was highlighted early in the pandemic. Quantitative detection of antibodies was considered an essential requirement to perform immunogenicity and efficacy studies and eventually to establish thresholds for protective correlates (8). However, standardization is necessary to allow comparability of quantitative antibody test results. Therefore, an international standard for SARS-CoV-2 antibodies (National Institute for Biological Standards and Control [NIBSC] 20/136) was issued by the WHO to compare SARS-CoV-2 specific antibody levels better (9). Although there is currently no general recommendation to determine antibody levels in all individuals after SARS-CoV-2 vaccination, this is reasonable from a scientific perspective and has been done in numerous studies (10–13). Moreover, it is now known that suboptimal or even lack of response to vaccination can occur in specific groups like immunocompromised patients (14, 15). These potential nonresponders might be identified in a first step by determining the antibody levels after vaccination. Unfortunately, there is little scientific evidence on the real-life comparability of different commercially available quantitative test systems, especially after vaccination (16, 17).

We could previously show that reporting standardized binding antibody units (BAU/mL) is insufficient for different test systems to provide numerically comparable results (16). Moreover, antibody responses are dependent on the type of vaccine used (18, 19). In this view, the temporal kinetics of antibody levels after vaccination were described for different vaccines and different antibody assays (20–23). However, the factors that may influence the comparability of different quantitative SARS-CoV-2 antibody tests have not been sufficiently systematically studied.

In the present work, we aimed to expand this knowledge using samples from AZD1222 vaccinated volunteers and tested antibody levels at multiple time points: 3 weeks after the first vaccine dose, 11 weeks after the first dose (immediately before the second dose), and 3 weeks after the second dose. Moreover, prebooster and postbooster levels were compared to SARS-CoV-2 specific T cell interferon γ responses. We used two of the most widely applied commercially available assays, the Roche Elecsys SARS-CoV-2 S-ECLIA (24) and the Abbott Anti-SARS-CoV-2 IgG II (25), to examine the comparability of the assays concerning the timing of blood collection after vaccination.

## RESULTS

### Agreement between Roche and Abbott assays depends on the timing of blood collection.

**FIG 1:**
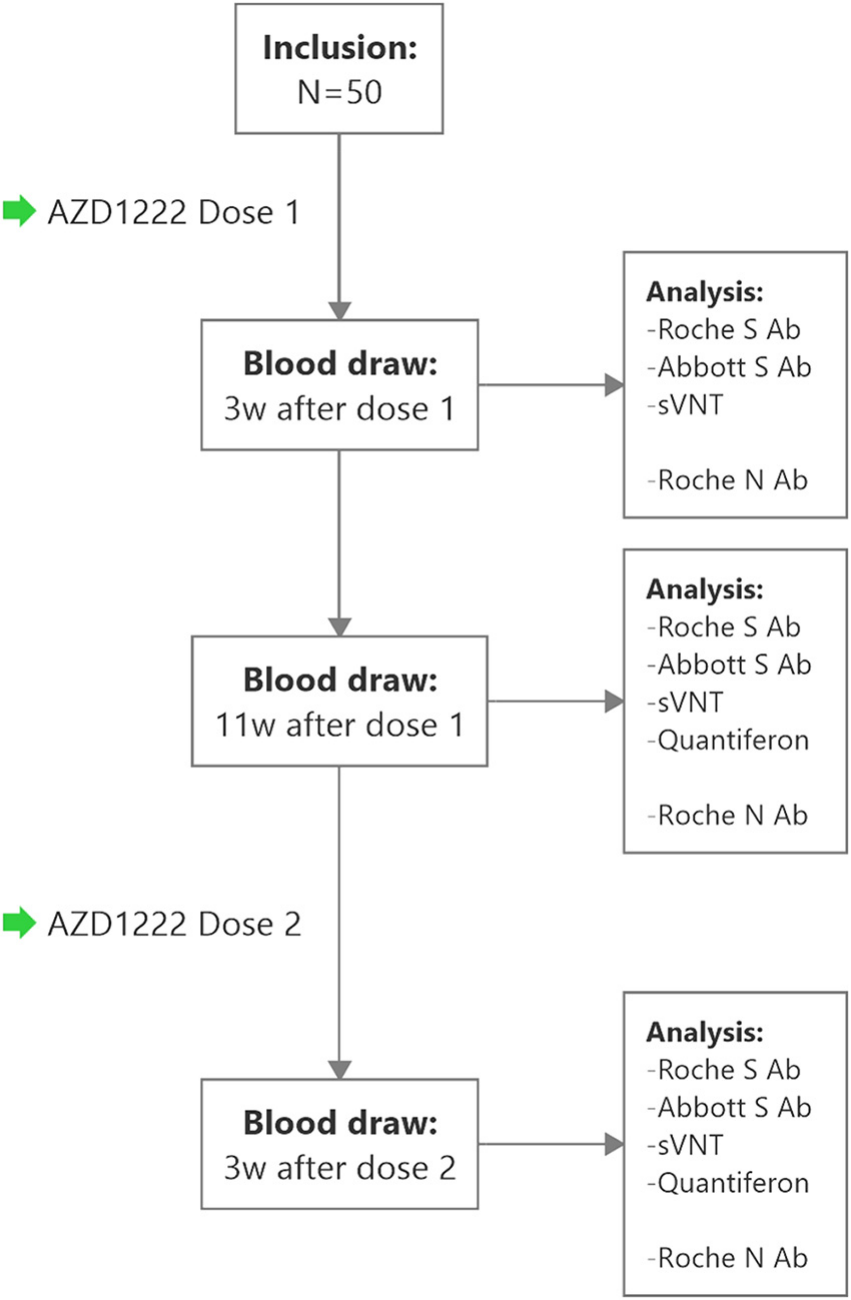
Study flow chart. Anti-Spike(S)-antibody (Ab) assays: Roche S, Abbott S. Infection with SARS-CoV-2 was ruled out by detection of antibodies against the SARS-CoV-2 nucleocapsid (N) using the Roche N ECLIA. W, weeks; sVNT, surrogate virus neutralization test.

**FIG 2:**
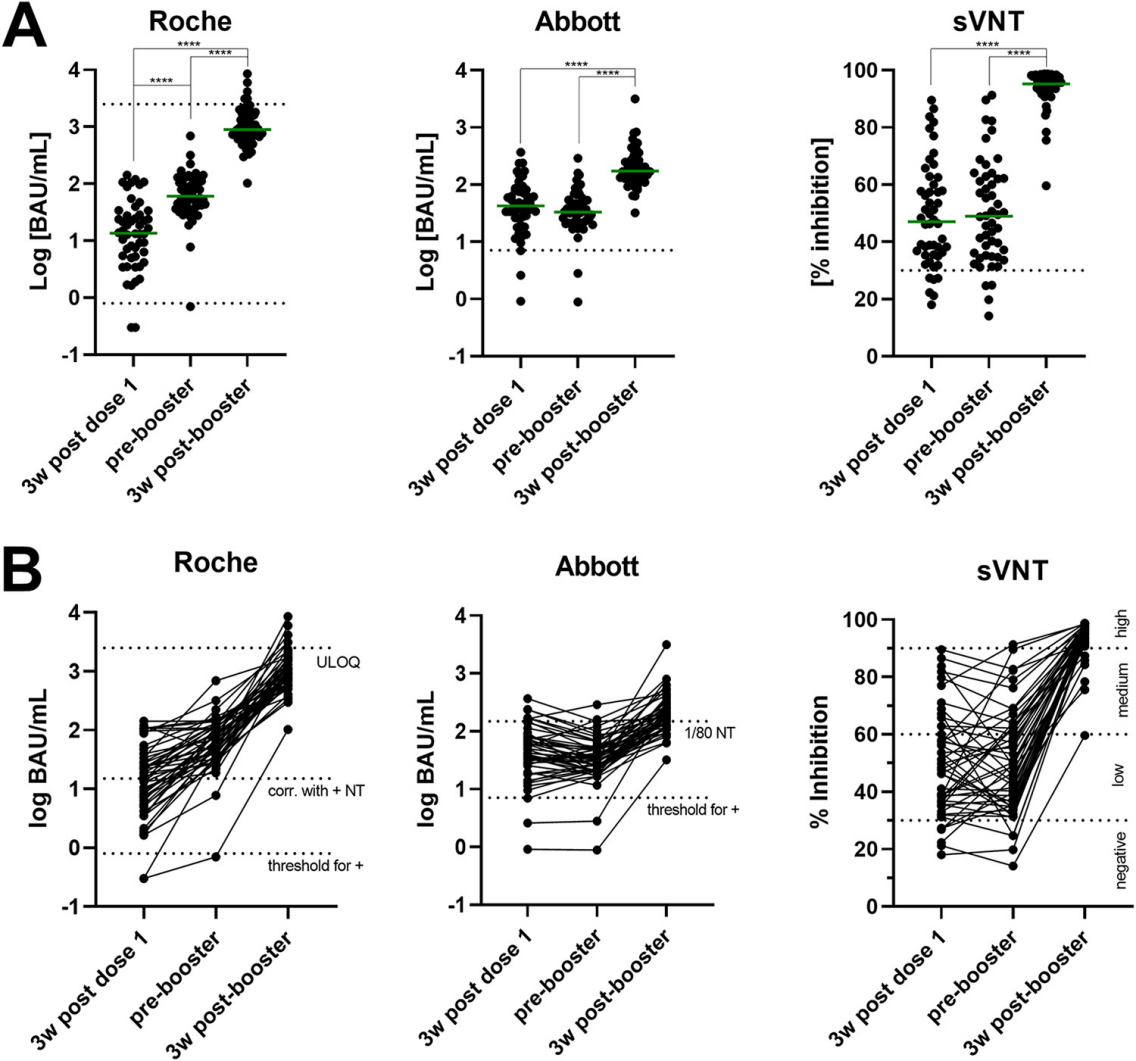
(A) Antibody levels (Roche, Abbott) and percent inhibition in a surrogate virus neutralization test (sVNT) 3 wk (weeks) after the first dose of AZD1222, prebooster (11w after the first dose), and 3 wk postbooster. Dotted lines indicate the test system threshold for positivity (Roche: 0.8 BAU/mL, Abbott 7.1 BAU/mL, sVNT 30%) and, in the case of Roche, the upper level of quantification (2,500 BAU/mL). Green lines represent the group median. ****, *P* < 0.0001 in Wilcoxon tests. (B) Longitudinal changes of individual Roche, Abbott, and cPass surrogate virus neutralization test (sVNT) results: 3 wk (3 weeks) after the first dose, before the booster dose, and 3 wk after the booster dose. According to the manufacturers, Roche results ≥15 BAU/mL correlate with a positive neutralization test, Abbott results ≥149,1 BAU/mL correspond to a neutralization titer of at least 1:80; 30% inhibition is considered the sVNTs threshold for positivity. Results are, according to the manufacturer, categorized into low (30 to 60%), medium (60 to 90%), and high (>90%) neutralizing capacity (all levels indicated by dotted lines).

Blood samples of 50 individuals were collected 3 weeks and 11 weeks after the first dose of AZD1222 (11 weeks = “prebooster”) and 3 weeks after the second dose (“postbooster”) (Fig. 1). Participant characteristics and binding assay levels at all assessed time points are presented in Table 1 and Fig. 2.

**TABLE 1:** Participant characteristics and surrogates of humoral (Roche, Abbott, sVNT) and cellular (IFN-γ) immunity^*a*^

Variable	Median (interquartile range) or counts (%)		
Female sex	43 (86%)		
Age [yrs]	35.5 (29 to 49)		
Immunosuppressive drugs	2 (4%)		
	Males (100%)	Females (100%)	
<30 yrs	1 (14%)	12 (28%)	
30–40 yrs	3 (43%)	11 (26%)	
41–50 yrs	2 (29%)	11 (26%)	
51–60 yrs	1 (14%)	8 (19%)	
>60 yrs	0	1 (2%)	
	3 wk post-dose 1	prebooster	3 wk postbooster
Roche [BAU/mL]	13.6 (5.2–29.9)	60.2 (36.4–112.8)****^b^	895.5 (611.8–1681.0)****,****
Abbott [BAU/mL]	42.2 (26.0–79.0)	32.9 (20.8–53.7)^**^	171.2 (123.4–278.7)****,****
sVNT [% inhibition]	47.1 (35.5–60.6)	49.0 (35.3–62.4)^ns^	95.2 (92.1–97.9)**^**,^****
IFN-γ Ag1-Nil [IU/mL]		0.10 (0.05–0.16)****^c^	0.15 (0.09–0.32)****,****
IFN-γ Ag2-Nil [IU/mL]		0.16 (0.09–0.31)****	0.31 (0.16–0.98)****,****

a BAU/mL, binding antibody units per milliliter; sVNT, surrogate virus neutralization test.

b All *P* values obtained from global Friedman tests were <0.0001. ns, not significant, **, *P* < 0.01, ****, *P* < 0.0001. For prebooster, *P* values are for post hoc comparisons with 3 wk post-dose 1 levels. For 3 wk postbooster, *P* values are for comparison with 3 wk post-dose 1 and prebooster levels (separated by comma).

c For Quantiferon, *P* values are for prebooster levels are for comparisons of the responses between time points (e.g., prebooster Ag1 versus postbooster Ag1) followed by the comparison of the responses to the two antigens at the same time (i.e., comparison of Ag1 with Ag2). ns, not significant, **, *P* < 0.01, ****, *P* < 0.0001.

In brief, Roche S antibody levels significantly increased from 13.55 BAU/mL (5.21 to 29.88) at 3 weeks after the first dose to 60.20 (36.38 to 112.80) directly before the booster (all *P* < 0.0001). Three weeks after the booster, the median levels were 895.50 (611.80 to 1681.00). With Abbott, results remained stable between 3 and 11 weeks after the first dose: 42.23 BAU/mL (26.00 to 78.99) and 32.88 BAU/mL (20.78 to 53.69), *P* = 0.178, and rose to 171.20 BAU/mL (123.40 to 278.70) 3 weeks after the booster (*P* < 0.0001). Similar to the Abbott test, the surrogate virus neutralization test (sVNT) did not show significant changes between week 3 and 11 after the first dose but significantly increased 3 weeks after the second dose: 47.1% inhibition (35.5 to 60.6), 49.0 (35.2 to 62.4), 95.2 (92.1 to 97.9); 3 wk postbooster versus prebooster or 3 wk after dose 1, *P* <0.0001. In terms of relative changes in antibody levels for individual participants when comparing 3 and 11 weeks after the first dose versus 3 weeks after the second dose, we observed a 14.2-fold (8.4 to 30.8) and 80.8-fold (27.4 to 191.0) change in titers for Roche, and a 4.9-fold (3.0 to 10.1) and 4.5-fold (2.2 to 9.9) change for Abbott. Thus, the Roche test discriminated increases in antibody levels between weeks 3 and 11 after the first dose: 4.7-fold (2.2 to 9.5) change between weeks 3 and 11, whereas Abbott did not with a 0.8-fold (0.5 to 1.4) change between weeks 3 and 11.

**FIG 3:**
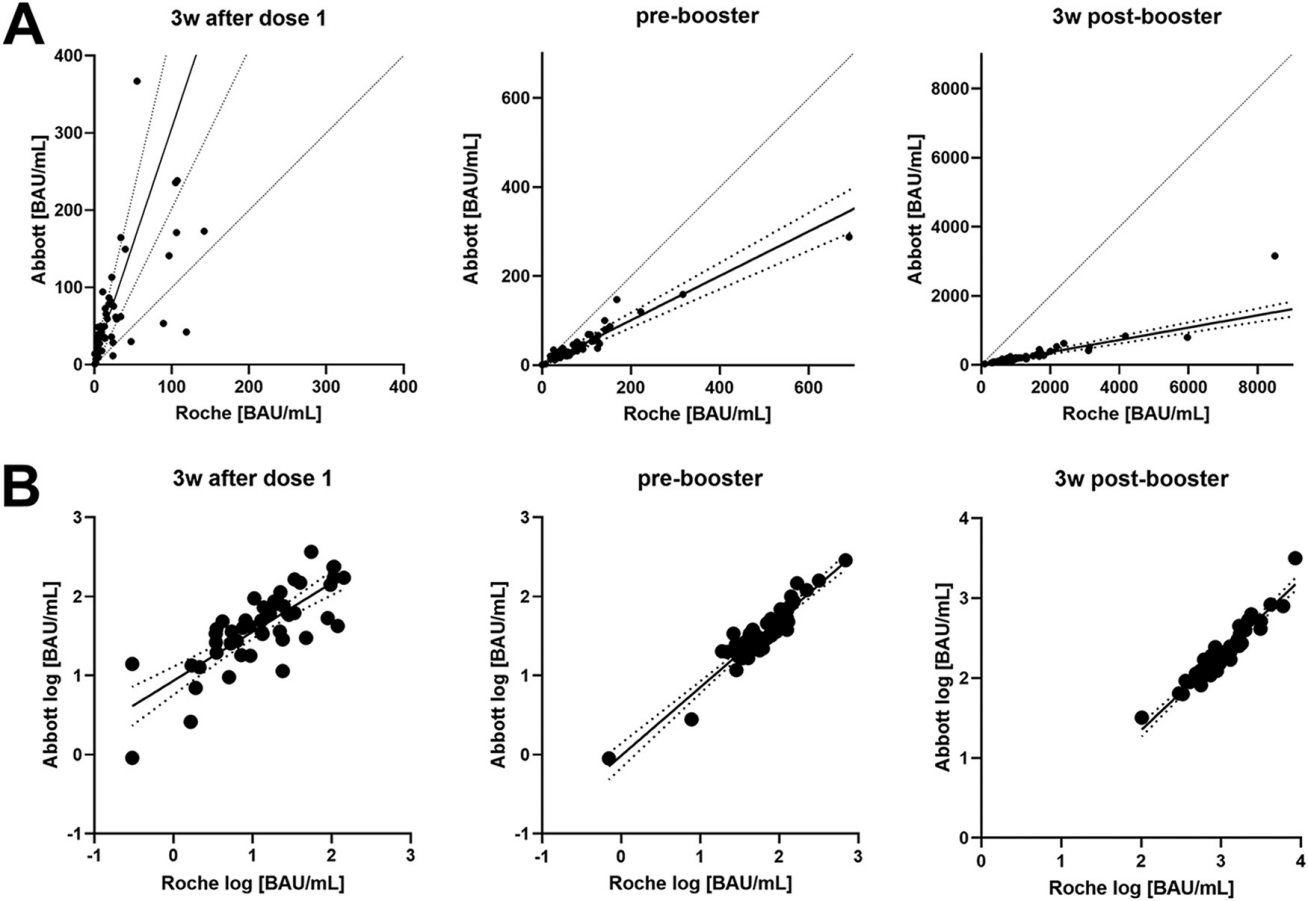
(A) Passing-Bablok regression for Roche and Abbott results; readings were converted to BAU/mL. The dotted lines are the 95% confidence intervals (CI) for the regression lines. The dashed lines represent lines of equality. (B) Linear regression (±95% CI) of logarithmic results from Roche and Abbott. 3 wk, 3 weeks; BAU/mL, binding antibody units per milliliter.

Three weeks after the first dose, results from Roche and Abbott binding assays showed a moderate correlation (ρ = 0.755, *P* < 0.0001). Passing-Bablok regression analysis revealed the equation Abbott = 7.4 + 2.99 × Roche, whereby only the slope of the equation was statistically significant (2.06 to 17); intercept: (−4.0 to 13.9). The agreement between both tests improved markedly 11 weeks after the first dose with the correlation coefficient rising to ρ = 0.902, *P* < 0.0001. Passing-Bablok regression revealed that BAU/mL derived from Abbott were approximately half those measured by Roche: Abbott = 1.1 + 0.50 × Roche (intercept: −2.4 to 5.5, slope: 0.43 to 0.56). Three weeks after the second dose, the correlation between Roche and Abbott remained excellent (ρ = 0.950, *P* < 0.0001). However, the conversion between the values changed again, with Roche values approximately 5 to 6 times higher than Abbott = 13.3 + 0.178 × Roche (intercept: −4.5 to 29.5; slope: 0.16 to 0.20) (Fig. 3).

There were no significant correlations between age and antibody levels at any distinct point in time. However, the response to the booster shot (BAU/mL_post-booster_ – BAU/mL_pre-booster_) showed a moderate correlation with age for both the Roche (ρ = 0.29, *P* = 0.042) and the Abbott (ρ = 0.29, *P* = 0.040) assay.

### Correlation between binding assay and sVNT results.

Next, we aimed to determine which of the two binding assays correlated better with neutralizing antibodies, particularly 3 weeks after the first dose, where the agreement between Roche and Abbott results was poorest. Neutralizing antibodies were estimated using the FDA-EUA approved (US)/ Conformitee Europeene - In-vitro diagnostic medical devices (CE-IVD) marked (Europe) cPass sVNT, with 30% inhibition as the threshold for positivity. Three weeks after the first dose, 44/50 (88%) participants yielded results above this threshold with a median inhibition of 47.1% (35.5 to 60.6). At week 11 directly before the booster, 4 of those with a negative result rose above 30% inhibition, but two with initially positive results decreased below the threshold, resulting in a total of 4 individuals (8%) below 30% inhibition. The median neutralizing capacity remained nearly unchanged at inhibition of 49.0% (35.2 to 62.4). Three weeks after the booster dose, all but one participant presented with at least medium neutralizing capacity (>60%; Fig. 2). The median increased to 95.2% inhibition (92.1 to 97.9) at week 3 post booster (Table 1 and Fig. 2B).

**FIG 4:**
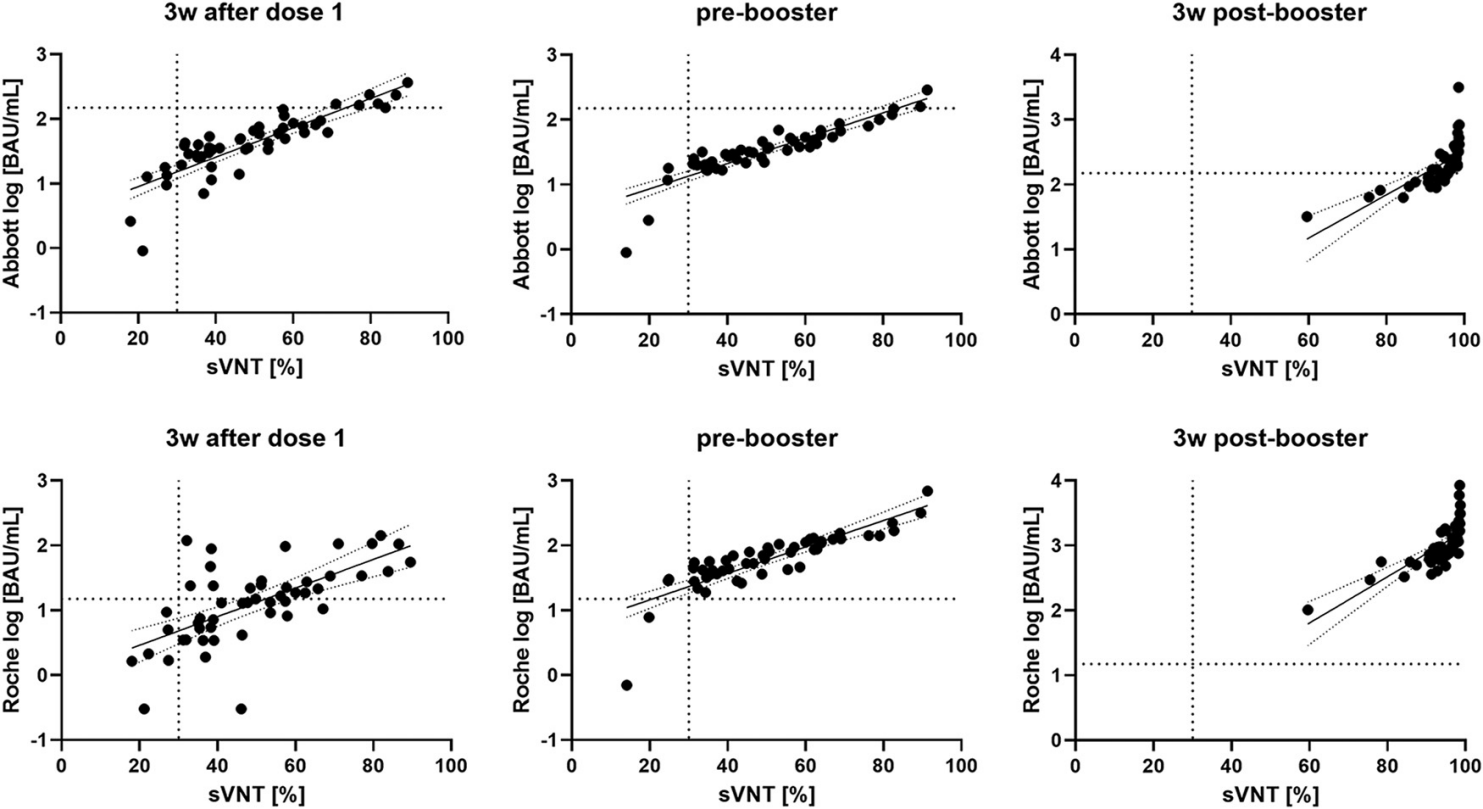
Linear regression lines (±95% confidence intervals) for c-pass surrogate virus neutralization test (sVNT) results and logarithmic binding assay results (top, Abbott; bottom, Roche). The dotted vertical line represents the sVNT threshold for positivity (30% inhibition). According to the manufacturers, Roche results ≥15 BAU/mL correlate with a positive neutralization test, Abbott results ≥149,1 BAU/mL correspond to a neutralization titer of at least 1:80 as indicated by horizontal dotted lines. 3 wk, 3 weeks; BAU/mL, binding antibody units per milliliter.

As shown in Fig. 4, sVNT percent inhibition at 3 weeks after the first dose correlated with the Abbott assay at ρ = 0.887, *P* < 0.0001. In contrast, the correlation with the Roche test was slightly lower at ρ = 0.666 (*P* < 0.0001). At 11 weeks after the first dose, sVNT results correlated very well with both assays (Abbott: ρ = 0.930, Roche: ρ = 0.894; both *P* < 0.0001). Similar results were observed at 3 weeks after the booster (Abbott: ρ = 0.877, Roche: ρ = 0.837; both *P* < 0.0001).

These data suggest that qualitative differences between early and late SARS-CoV-2 antibodies may affect the comparability of serological tests.

### Relative changes of T cell responses, but not absolute IFN-γ levels, correlated with antibody levels.

**FIG 5:**
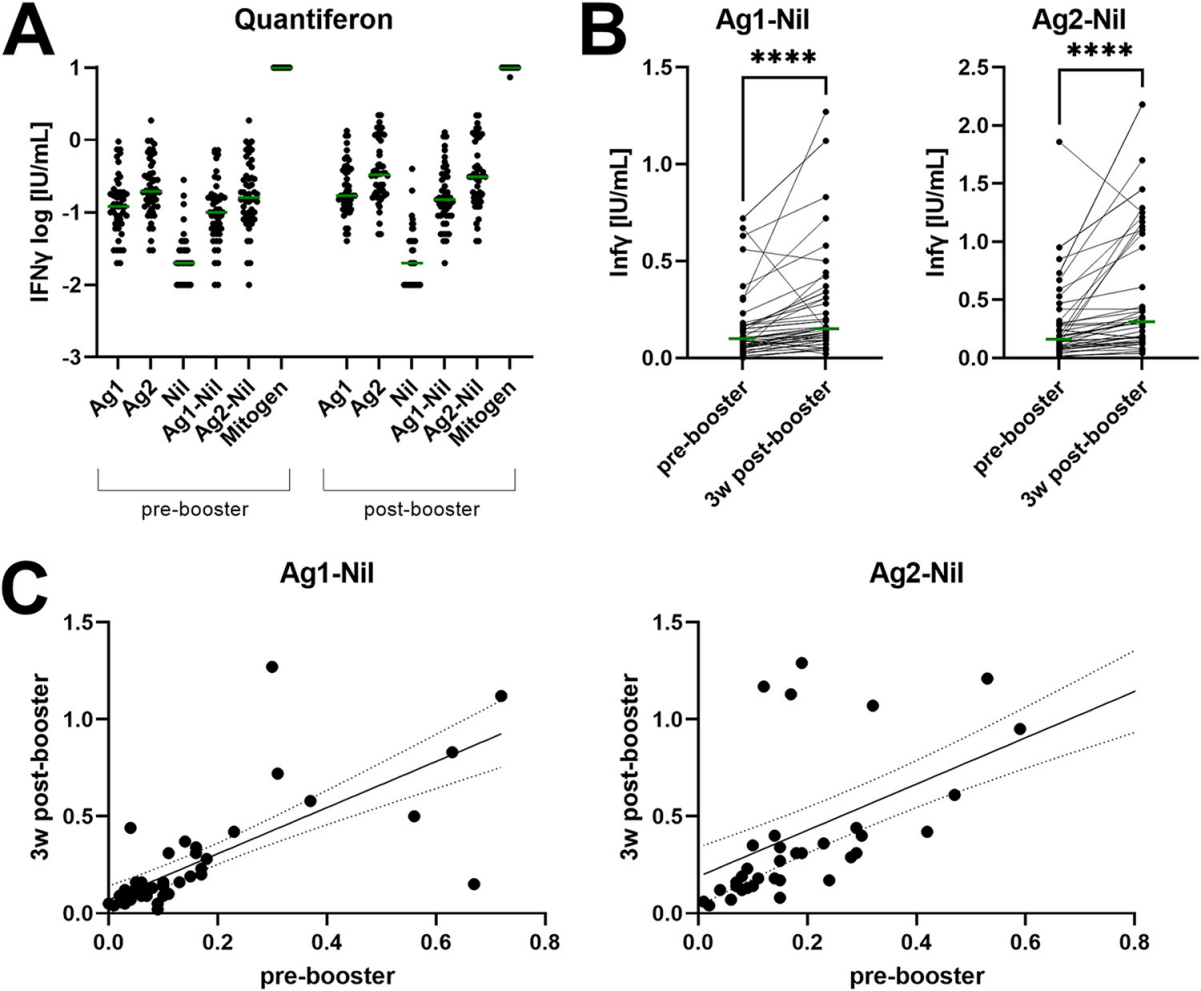
(A) Logarithmic IFN-γ levels after stimulation of 1 mL heparinized whole blood with Quantiferon SARS-CoV-2 antigen mixture 1 (Ag1), antigen mixture 2 (Ag2), Nil (negative control), and mitogen control, as well as Nil-corrected levels (Ag1-Nil, Ag2-Nil). Green lines indicate medians. (B) Pairwise comparisons of Ag1-Nil and Ag2-Nil in response to the booster shot. **** <0.0001. Green lines indicate medians. (C) Linear regression curves (±95% confidence intervals) of Ag1-Nil and Ag2-Nil before and after the booster shot. 3 wk, 3 weeks.

Finally, we examined the interactions between T cell and antibody responses (quantified with the Abbott and the Roche test). For this purpose, we compared the changes between the time before the booster (11 wk after the first dose) and the time after the booster (3 wk after the second dose), as the antibody response to the booster shot is by far more pronounced than that to dose 1, allowing larger effect sizes to be compared. Interferon-γ (IFN-γ) response to both used antigen mixtures (Ag1 and Ag2) increased after the booster shot: Ag1 negative (Ag1-Nil) 0.10 IU/mL (0.05 to 0.16) to 0.15 IU/mL (0.09 to 0.32), *P* < 0.0001; Ag2-Nil 0.16 IU/mL (0.09 to 0.31) to 0.31 IU/mL (0.16 to 0.98) (Fig. 5 and Table 1). Levels from both antigen mixtures correlated well with each other (prebooster ρ = 0.725, *P* < 0.0001; postbooster ρ = 0.775, *P* < 0.0001; Fig. 5). Moreover, prebooster levels were in good agreement with postbooster levels (Ag1-Nil ρ = 0.786, *P* < 0.0001; Ag2-Nil ρ = 0.832, *P* < 0.0001).

Prebooster IFN-γ levels were only weakly correlated with antibody levels. Most correlations lacked statistical significance, and no relevant correlation was found 3 weeks after the booster. In contrast, the relative changes of cellular and binding assay antibody responses, calculated as 100 × (postbooster – prebooster)/prebooster, correlated significantly after incubation with Ag2. However, for Ag1, statistical significance could not be reached (Table 2). Interestingly, no such correlation was observed for the sVNT.

**TABLE 2:** Spearman’s ρ of rank correlations between Nil-corrected interferon γ (IFN-γ) levels after incubation with Quantiferon SARS-CoV-2 antigen mixtures 1 (Ag1-Nil) or 2 (Ag2-Nil) and antibody levels^*a*^

Variable	Abbott	Roche	sVNT
Prebooster			
Ag1-Nil	0.236 (*P* = 0.100)	0.258 (*P* = 0.071)	0.217 (*P* = 0.130)
Ag2-Nil	0.287 (*P* = 0.044)	0.243 (*P* = 0.089)	0.252 (*P* = 0.077)
Postbooster			
Ag1-Nil	0.037 (*P* = 0.796)	0.101 (*P* = 0.485)	−0.001 (*P* = 0.995)
Ag2-Nil	0.067 (*P* = 0.642)	0.095 (*P* = 0.510)	0.024 (*P* = 0.868)
% response			
Ag1-Nil	0.251 (*P* = 0.082)	0.247 (*P* = 0.087)	0.196 (*P* = 0.178)
Ag2-Nil	0.333 (*P* = 0.018)	0.325 (*P* = 0.021)	0.164 (*P* = 0.255)

a sVNT, surrogate virus neutralization test. % response, 100 × (postbooster – prebooster)/prebooster.

## DISCUSSION

SARS‐CoV‐2 specific anti‐spike protein assays have been and are still widely used for serological studies. While the distinction between positive and negative is usually sufficient in seroprevalence studies, quantitative results are needed to describe the immunogenicity of SARS-CoV-2 vaccines and, ideally, to find protection correlates (13, 26). However, the quantitative results of different SARS-CoV-2 antibody tests must be comparable to summarize the results of different studies or to translate them to other situations (8, 9). In the present work, we compared two commercially available and broadly used CE-IVD marked SARS-CoV-2 antibody assays (Roche and Abbott). Both assays quantitate antibodies directed against the RBD domain of the SARS-CoV-2 spike protein and were referenced against the first WHO standard for SARS-CoV-2 antibodies, thus providing results in BAU/mL. We demonstrated in a previous study after vaccination with BNT162b that despite the standardization of SARS-CoV-2 antibody assays, the numerical values of different test systems are not interchangeable (16). Now we show for the first time that the problem of comparability is even more complex because the conversion between different assays can change dramatically with the time interval from vaccination.

From studies with convalescents, we know that the levels of antibodies depend on the test system used and that, in addition, other differences may become observable over time, e.g., levels appear to decline more rapidly with one test than with another (27). However, these findings cannot be transferred to the situation after vaccination without restrictions. There are known differences between the serostatus after infection and vaccination (28), and the various vaccines also differ in this respect (29).

Using samples from 50 individuals vaccinated with AZD1222, we could show that both anti-spike antibody assays used (Abbott and Roche) detected specific antibodies in all but two participants 3 weeks after the first dose. Both nonresponders were taking immunosuppressive drugs, which can lead to a decreased to absent response to the vaccine (30–32). However, after the second dose, the antibody levels markedly increased and reached detectable levels in all participants (Table 1 and Fig. 2A). In contrast to people with previous COVID (33–35), the second dose was required in our SARS-CoV-2 naive population to induce high antibody levels. The median relative change of individual antibody levels 3 weeks after the first versus 3 weeks after the second dose was nearly 20-times higher for Roche than for Abbott (80.8 versus 4.5-fold change; Fig. 2B). Despite targeting the same antigen (RBD) and reporting in BAU/mL, not even the relative increases in antibody levels turned out to be comparable. Of course, it should be mentioned that the exact antigens used in the two test systems are unlikely to be identical, and therefore such differences may be due to different epitope specificities. Furthermore, Abbott and Roche differ in the test format where the former uses classical IgG-specific detection, whereas the latter applies a sandwich format to bind all antibody isotypes potentially. The limited comparability of serological assays after vaccination is not specific for the AstraZeneca vaccine, as it was also observed after immunization with Pfizer/BioNTech BNT162b2 (16).

When looking at the difference between 3 and 11 weeks after the first vaccination dose, it was found that both the Abbott test and the cPass sVNT did not detect an increase in antibodies (Fig. 2). In contrast, antibody levels measured by the Roche test increased >4-fold during this period. This discrepancy could be either explained by the inability of the Abbott and sVNT assays to distinguish such small changes in antibody concentration or the possibility that the Roche assay detects not only quantitative but also qualitative changes of the antibodies formed. Because previous studies failed to demonstrate a continuous increase in antibody levels for AZD1222 later than 3 weeks after vaccination, the Roche total antibody sandwich assay may also be sensitive to qualitative changes in nascent antibodies (e.g., antibody maturation), in contrast to the Abbott IgG-specific assay (12, 36). This hypothesis is also supported by the observation that in direct comparison with the sVNT, the Roche assay underestimated inhibitory capacities at week 3 (Fig. 4), which is discussed in detail below. Thus, the assays studied show significant differences in the kinetics of antibody levels, which has been reported previously but was only rarely demonstrated from the same sample with different assays (17, 37). Although the correlation between Roche and Abbott improved over time, their relationship changed significantly depending on the time of blood sampling (Fig. 3). At the first time point, Roche measured three times lower values in BAU/mL than the Abbott assay, 11 weeks after the first dose, Roche measured twice as high as Abbott, and finally, after the booster, Roche was median 5 to 6 times higher than Abbott. Currently, further evidence is lacking on whether the association between two antibody tests can become constant and at what time interval to the second dose this would be the case. But it should also be considered that each new stimulus of antibody formation would lead to different ratios. Considering the ongoing administration of a third dose and already starting vaccination of fourth doses, continuous ratios changes would be likely. It is important to note that, unlike other comparative (often retrospective) studies, we did not choose time intervals but clearly defined time points in this prospective observational study. Therefore, an accurate assessment of the observed time-dependent effects was possible, which could otherwise be overlooked.

As studies have shown before that detection of SARS-CoV-2 anti-spike binding antibodies correlates well with the presence of functional neutralizing antibodies, we wanted to examine differences between Roche and Abbott assays in this regard (38–43). The agreement between the results of the binding antibody assays and the neutralization test surrogate was generally good (Fig. 4). At 11 weeks after the first vaccination, the correlation was excellent; after the booster, the correlation was technically limited due to many participants reaching the plateau of the sVNT. However, the worst correlation was found for the first antibody response 3 weeks after the first dose, and here Roche performed significantly worse than Abbott. This finding may be important because the improvement in Roche/sVNT correlation from ρ = 0.666 to ρ = 0.894 between 3 and 11 weeks after the first dose may indicate reduced sensitivity of the Roche assay for early antibodies. In other words, the discrepancy mentioned above that only Roche showed increasing antibody levels between 3 and 11 weeks after the first dose, while the other two tests showed identical or even slightly decreasing levels (Fig. 2), could mean that the Roche test requires more matured antibodies to allow binding.

In line with previous studies, the second dose of AZD1222 substantially enhanced the initial antibody response in our cohort (19, 36, 44). Because the formation of antibodies is physiologically supported by specific T cells, we assumed a correlation between T cell response and antibody formation. Therefore, we aimed to additionally investigate the cellular responses elicited by the second dose. For this purpose, we used a SARS-COV-2 Quantiferon IFN-γ release assay similar to those known from tuberculosis diagnostics and compared the time points before and after the second vaccine dose. As previously shown (45), the second dose induced an increase in cellular reactivity in most cases (Fig. 5). Three participants presented negative responses to Ag1 and Ag2, but the decreases were only moderate (0.04 to 0.07 IU/mL). The antibody responses in these participants were comparable to those seen in the rest of the cohort. However, we found only weak, mostly statistically nonsignificant correlations between antibody levels and absolute IFN-γ levels before the second dose and no correlation at all after the second dose (Table 2).

In contrast, the percent cellular response (fold change) to the second dose correlated significantly with the percent antibody response (ρ = 0.33 for both binding assays), see Table 2. This finding suggests that the increase of antibodies after a second shot, which is detected by both binding assays, can be substantiated by an accompanying cellular reaction. In contrast, we found no correlation between the relative changes in cellular and sVNT response, which might be partly explained by the limited measurement range of the sVNT. However, because not all antibodies formed are functionally active neutralizing antibodies (NAbs), even not all of those specifically directed against the receptor-binding domain (RBD) of the spike protein, the binding antibodies may be superior to the measurement of NAbs here as a correlate for cellular activation.

This study has several strengths and limitations: although 50 participants might be considered a relatively small cohort, we have shown in previous work that this number is sufficient for such comparative approaches and that our data could be replicated in much larger cohorts (16, 46). One strength of our study is that we followed exact time points for blood sampling in the context of a prospective observational study. A limitation is that we did not perform measurements beyond 3 weeks after the second dose. Thus, whether a constant ratio would be observed after a specific time is unclear. Furthermore, our cohort using AZD1222 (inducing significantly lower median antibody levels than, e.g., BNT162b2) has the advantage of a broader distribution of values across the measurable spectrum with a very low proportion of results above 1,000 BAU/mL. As this value represents the upper limit of referencing with the WHO SARS-CoV-2 antibody standard, a linear relationship is no longer guaranteed for values above this, leading to unwanted biases in comparing different antibody tests.

In summary, with the present work, we show for the first time that the comparability of quantitative anti-spike SARS-CoV-2 antibody tests is highly dependent on the timing of blood collection. Although the results of the two assays studied correlate well at all time points, a numerical agreement is only possible through a correction factor, which changes over time after both the first dose of vaccine and the stimulus of the second dose of vaccine. Concerning boosters with a 3rd and possibly fourth dose, we must assume that the relationship between two antibody assays may be in a constant state of change. Therefore, it does not seem feasible to compare different quantitative SARS-COV-2 antibody results without standardization of the time of sample collection.

## MATERIALS AND METHODS

### Study design and participants.

We included sera of 50 participants in this prospective observational performance evaluation study. Inclusion criteria were an age ≥18 years and willingness to donate blood during the MedUni Wien Biobank’s healthy blood donor collection (Medical University of Vienna ethics committee vote no. 404/2012). Incomplete follow-up samples and seropositivity for anti-nucleocapsid antibodies due to infection with SARS-CoV-2 lead to exclusion. The study protocol was reviewed and approved by the Medical University of Vienna ethics committee (1066/2021) and conforms with the Declaration of Helsinki.

### Laboratory methods.

Blood samples were taken 3 weeks and 11 weeks (“prebooster”) after the first dose of AZD1222, as well as 3 weeks after dose 2. At prebooster and postbooster time points, an additional amount of 4 mL blood was drawn to estimate T cell immunity (Fig. 1). Blood samples were processed and stored according to standard operating procedures by the MedUni Wien Biobank in an ISO 9001:2015 certified environment (47).

Previous SARS-CoV-2 infection was assessed by the Roche Elecsys SARS-CoV-2 nucleocapsid ECLIA (Roche, Rotkreuz, Switzerland) on Cobas e801 modular analyzers (Roche) (48). This assay detects total antibodies against the viral nucleocapsid, which are induced after infection, but not after vaccination with AZD1222. To not miss a previous infection by antibody waning, the response to the first dose was monitored; convalescent vaccinees usually react with a pronounced antibody response to dose 1 that is comparable to the response to dose 2 in naive individuals (33).

Vaccine-induced antibodies against the viral spike protein were quantified using the Roche Elecsys SARS-CoV-2 S-ECLIA (49) and the Abbott Anti-SARS-CoV-2 IgG II (50). This Roche test is a quantitative (range: 0.4 to 2,500 BAU/mL, whereby the conversion factor of the system’s arbitrary U/mL to BAU/mL is 1.0) total antibody sandwich assay recognizing antibodies directed against the receptor-binding domain (RBD) of the SARS-CoV-2 spike (S) protein and was performed on Cobas e801 modular analyzers (Roche), samples >0.8 BAU/mL are considered diagnostically positive. As a deviation from the product manual, samples exceeding the quantification range were manually prediluted at a dilution factor of 1:10. The Abbott assay also quantifies anti-RBD specific IgG-antibodies (range:1.0 to 11,360.0 BAU/mL, whereby the arbitrary AU/mL have been converted to BAU/mL by multiplying them with 0.142) and was applied on an Abbott Architect i2000r (Abbott, USA). The assay’s threshold for positivity is 7.1 BAU/mL, which equals 50 AU/mL.

Binding reactivities (Roche, Abbott) were compared to a well-described CE-IVD marked surrogate virus neutralization test (GenScript cPass sVNT) (51, 52). This sVNT quantifies the serum’s ability to inhibit spike/ACE-2 interaction. Results with an inhibition >30% are considered positive.

The T cell activity was estimated in prebooster and postbooster whole blood samples using the Quantiferon SARS-CoV-2 assay (Qiagen, Hilden, Germany [53–55]). In brief, we quantified the IFN-γ-release after a 21 h incubation of 1 mL heparinized whole blood portions with two different SARS-CoV-2 antigen mixtures (Ag1, Ag2), and with a negative (“Ce point, IFN-γ) values of the negative (Nil) control were subtracted from the SARS-CoV-2 specific antigen mixes results of the samples and presented as Ag1-Nil and Ag2-Nil, respectively. Available literature suggests a cutoff around 0.15 to 0.20 IU/mL for detecting a specific response (54). Recently, the technically identical test has become available as an IVD certified test, and the manufacturer recommends a cutoff for positivity of ≥0.15.

### Statistical analysis.

Continuous data are presented as median (interquartile range), and categorical data as counts (percentages). Paired data are compared by Wilcoxon and Friedman tests. Correlations are calculated according to Spearman. Serological assays are compared by Passing-Bablok regressions, which assess differences between two test systems by estimating the slope (systematic proportional differences) and the intercept (systematic constant differences) of a linear regression line. There are no preconditions regarding the distribution of the measured values and the measurement errors to be met. *P* values <0.05 were considered statistically significant. All calculations have been performed using MedCalc 19.7 (MedCalc Bvba, Ostend, Belgium) and SPSS 26 (IBM, Armonk, USA). Graphs were drawn with Prism 9 (GraphPad, La Jolla, USA).
